# A ‘Frugal’ EGFET Sensor for Waterborne H_2_S

**DOI:** 10.3390/s24020407

**Published:** 2024-01-09

**Authors:** Zahrah Alqahtani, Martin Grell

**Affiliations:** 1 Physics Department, Faculty of Science, Taif University, P.O. Box 11099, Taif 21944, Saudi Arabia; 2 Llyfrgell Bangor, Ffordd Gwynedd, Bangor LL57 1DT, UK; martin@spinne.plus.com

**Keywords:** water, hydrogen sulphide, gold, EGFET, sewage

## Abstract

Hydrogen sulphide (H_2_S) is a toxic gas soluble in water, H_2_S_aq_, as a weak acid. Since H_2_S_aq_ usually originates from the decomposition of faecal matter, its presence also indicates sewage dumping and possible parallel waterborne pathogens associated with sewage. We here present a low footprint (‘frugal’) H_2_S_aq_ sensor as an accessible resource for water quality monitoring. As a sensing mechanism, we find the chemical affinity of thiols to gold (Au) translates to H_2_S_aq_. When an Au electrode is used as a control gate (CG) or floating gate (FG) electrode in the electric double layer (EDL) pool of an extended gate field effect transistor (EGFET) sensor, EGFET transfer characteristics shift along the CG voltage axis in response to H_2_S_aq_. We rationalise this by the interface potential from the adsorption of polar H_2_S molecules to the electrode. The sign of the shift changes between Au CG and Au FG, and cancels when both electrodes are Au. The sensor is selective for H_2_S_aq_ over the components of urine, nor does urine suppress the sensor’s ability to detect H_2_S_aq_. Electrodes can be recovered for repeated use by washing in 1M HCl. Quantitatively, CG voltage shift is fitted by a Langmuir-Freundlich (LF) model, supporting dipole adsorption over an ionic (Nernstian) response mechanism. We find a limit-of-detection of 14.9 nM, 100 times below potability.

## 1. Introduction

Hydrogen sulphide (H_2_S) is a colourless, water-soluble gas with the odour of rotten eggs that emanates from decomposing organic material, *e.g.*, in wastewater or as a byproduct of farming, or petroleum refining [1,2,3,4]. H_2_S has various detrimental effects both as a gas, and in aqueous solution (H_2_S_aq_), where it behaves as a weak acid, H_2_S_aq_ + H_2_O ⇋ HS^−^ + H_3_O^+^ (pK_a_ = 7.04). As a gas, it is toxic to humans and animals on inhalation [5,6], and corrosive in industrial processes such as syngas separations [7,8]. When ingested in aqueous solution it causes various diseases such as liver cirrhosis and Alzheimer’s disease [9].

H_2_S_aq_ is not only toxic itself, but as it originates in the decomposition of faecal matter, it is also a ‘telltale’ sign of sewage pollution, warning of the parallel presence of pathogens like typhoid fever or cholera bacteria associated with sewage [10]. In 2022, in England and Wales alone, water companies reported more than 384,000 discharges of raw sewage into watercourses, or the sea [11]. There is therefore a strong case for an affordable and widely available sensor for H_2_S_aq_, which is addressed in the present work.

The World Health Organisation (WHO) does not strictly define a ‘potability’ (maximum concentration, c_pot_, to accept as drinking water), but recommends that no water should be consumed without prior treatment if it has a noticeable ‘rotten egg’ odour. This is a somewhat vague recommendation as the human sense of olfaction may differ between individuals and ethnic groups, olfaction may be ‘numbed’ by prolonged exposure to H_2_S, and vapour pressure above an aqueous solution will depend on water temperature. These uncertainties underscore the demand for an objective sensor. We here follow the (clearly approximate) guideline given by WHO that human olfaction detects H_2_S odour in the headspace above water when the concentration of H_2_S_aq_ exceeds 1.5 μM [10] and use 1.5 μM as potability for H_2_S_aq_. We adopt this c_pot_ as a performance target for the limit-of-detection (LoD) of H_2_S_aq_ sensors.

Due to the environmental and health hazards of H_2_S, its detection both in air and in water has attracted significant attention. Traditional analytical methods include gas chromatography (GC) [12,13,14], electrochemical analysis [15,16], metal-induced sulphide precipitation [17,18,19], plasma-atomic emission [20], and surface-enhanced Raman scattering [21,22]. An innovative approach to H_2_S gas sensing is based on organic field effect transistors [23,24]. Often, the detection of H_2_S_aq_ relies on prior acidification of the sample to drive H_2_S_aq_ out of solution and subsequent quantitative gas chromatography. Commercial equipment for the detection of H_2_S_aq_ follows this procedure [25].

However, these methods require qualified operators, and complex, expensive, and stationary instrumentation. Communities affected by sewage discharge will benefit most from more ‘frugal’ sensor technologies. We understand ‘frugal’ as accessible in terms of both the cost of the required components and instruments, and the skills required of the sensor operators. Fluorescent H_2_S chemosensors have a lighter experimental footprint [26], but still require multi-step synthetic routes for the dye, and fluorescence spectroscopy instrumentation. Kim *et al.* [27] have reported a colourimetric (absorption-based) squaryllium dye that allows naked-eye detection of H_2_S gas, however, quantitative detection of H_2_S_aq_ with the same dye still requires optical instrumentation and an auxiliary solvent. Also, the squaryllium dye is not readily available but requires bespoke synthesis.

**Figure 1:**
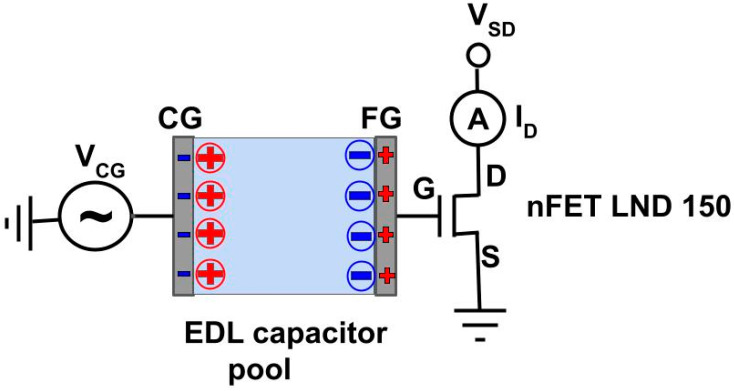
Schematic of an EGFET as used here. The blue shaded area represents water in the EDL capacitor pool. Charged electrode surfaces are shown as −/+, cations/anions as ⊕/⊖. The ionic layers facing the charged electrodes are known as Helmholtz layers.

Here, we report a field effect transistor (FET)-based potentiometric sensor for H_2_S_aq_ sensing. Our approach is based on stock electronic components and widely and cheaply available materials and equipment only. We require no dye, no optical equipment, and no chemical treatment to introduce a sensitiser, and there is no need to drive H_2_S_aq_ out of the solution first. As transducer, we use the ‘extended gate field effect transistor’ (EGFET) concept, first introduced by van der Spiegel *et al.* [28], which is a development of the original ‘ion-sensitive field effect transistor’ (ISFET) introduced by Bergveld [29]. The EGFET is the most ‘user-friendly’ potentiometric field effect transducer, as it separates the sensitive element from the transistor itself with the help of an electric double layer (EDL) capacitor, as described in ‘Experimental’ (Figure 1). The experimenter can work with an unmodified stock device, here the LND150 n-channel depletion mode (‘normally on’) FET [30]. 

To introduce sensitivity to H_2_S_aq_, we use the known affinity of sulphur compounds to gold (Au) surfaces. While gold is generally known to be chemically inert, organic thiols (R-SH) and similar sulphur compounds do readily self-assemble onto Au surfaces by adsorption of thiol to Au, as reviewed, *e.g.*, by Love *et al*. [31]. This is widely used for the immobilisation of biosensitisers (enzymes, antibodies, and similar proteins) to Au electrodes, reviewed, *e.g.*, by Zuilhof [32]. A typical example is cysteamine, H_2_N-C_2_H_4_-SH, which first binds to an Au electrode via its thiol (-SH) group, and then may condense at its amine (-NH_2_) group with an activated carboxyl group of a biosensitiser, *e.g.*, an immunoglobulin [33]. Here we show that affinity between thiols and Au extends to H_2_S, which can be seen as a thiol, R-SH, with R = H. We use this affinity to demonstrate a novel sensor for H_2_S_aq_: Immersing an Au wire as an electrode into an EDL capacitor pool filled with water containing H_2_S_aq_ leads to a surface potential without any further sensitisation. The potential is readily transduced by an EGFET. Calibration of the magnitude of potentiometric response *vs.* H_2_S_aq_ concentration establishes a simple EGFET sensor for H_2_S_aq_ with a limit-of-detection (LoD) well below potability.

## 2. Materials and Methods

### 2.1. EGFET Setup

We here used an EGFET setup, shown schematically in Figure 1.

Two electrodes, ‘control gate’ (CG) and ‘floating gate’ (FG) are immersed into an electric double layer (EDL) capacitor pool. Sometimes an electrochemical reference electrode is used as CG, but not here. The pool is filled with water which contains ions due to autoprotolysis (2H_2_O ⇋ OH^−^ + H_3_O^+^), and dissolved salts, thus forming an electrolyte. The water may also carry an *a priori* unknown concentration of an analyte, which is the target of EGFET sensors. As FET, we here use a n-channel depletion mode (‘normally on’) MOSFET type LND150. The LND150 is not purpose- built for EGFET sensors, but is manufactured for generic electronic engineering purposes like high voltage switches, precise constant current sources, and voltage ramp generators [30] by several manufacturers, including Supertex Inc. (Sunnyvale, CA, USA) and MICROCHIP (Chandler, AZ, USA). However, its ‘normally on’ property, affordable price (<1 £/unit) and well documented and consistent performance characteristics [30] have made it somewhat of a default choice for EGFET applications, *e.g.*, [33,34,35]. A voltage from source *V_CG_* is not directly applied to the LND150 gate, but to CG. *V_CG_* is communicated across the pool to the LND150 gate via a pair of EDLs, *i.e.*, interfacial double layers (‘Helmholtz layers’) that form at the electrode/electrolyte interfaces. In Figure 1, these are shown for *V_CG_* < 0, with a negatively charged CG electrode surface ‖ cationic Helmholtz layer in the adjacent electrolyte, and anionic Helmholtz layer ‖ positively charged electrode surface in the adjacent FG electrode. The order is reversed for *V_CG_* > 0. FG is connected to the FET gate, communicating *V_CG_* to LND150 gate. However, LND150 gate voltage *V_G_* differs from *V_CG_* by any potential that develops within the EDL pool:

*V_G_* = *V_CG_* + *V_Pool_* (1)

EGFET sensors usually operate by deliberately introducing a ‘sensitiser’ (aka ‘receptor’) into the EDL pool that binds to a target analyte and develops an analyte-concentration dependent potential. However, in some cases [34], as well as here, the surface of an electrode itself may act as sensitiser.

### 2.2. EGFET Electric Characterisation

For EGFET characterisation, we used a source-measure unit (SMU) model number P2005A2 from Ossila, Sheffield, UK, to sweep *V_CG_* in steps of 32 μV, covering a range of ~3 V around zero but largely covering negative *V_CG_* corresponding to the negative threshold of the ‘normally on’ LND150. After every step, we allowed the EGFET to settle for 0.6 s, then measured the resulting LND150 drain current at V_SD_ = +0.2 V with another Ossila SMU, giving the transfer characteristic *I_D_*(*V_CG_*). When maximum *V_CG_* was reached we reversed the gate voltage sweep direction to record transfers over a full hysteresis loop. All transfers shown in this work display *I_D_* over a full *V_CG_* loop, *i.e.*, there are two values for *I_D_*, one at rising/another at falling *V_CG_*. However, hysteresis was mostly absent, sometimes a small hysteresis was observed, giving the impression of a single *I_D_*(*V_CG_*) curve in most cases.

### 2.3. Sensitising EGFET for H_2_S

An EGFET can be sensitised to target ions by immobilising a sensitiser on either electrode or in a membrane that divides the pool. If the binding of the target analyte to sensitiser leads to an interface or membrane potential, *V_Pool_* in Equation (1) changes and the transfer characteristic shifts along the *V_CG_* axis. Here we did not sensitise via a membrane or immobilised sensitiser, but tested gold (Au) electrode surfaces for innate sensitivity to H_2_S as the analyte, as proposed in the introduction. As the ability of electrodes to communicate via electric double layers is rather independent of their size, we used rather small electrodes to minimise the demand for gold, namely 2 cm (length) × 0.5 mm (diameter) of 99.95% pure gold wire as H_2_S-sensitive electrode, and 2 cm (length) × 1 mm (diameter) of 99.999% pure zinc wire as an electrode that is presumably insensitive to H_2_S. Au and Zn wires were sourced from Goodfellow and Sankuai, respectively. We used these Au and Zn electrodes as CG/FG electrodes in an EDL pool, *cf.* Figure 1, in different permutations, as listed in Table 1 below, in a pool containing (5…6) mL of water.

### 2.4. EGFET H_2_S Sensor Testing and Calibration

As a realistic background ‘matrix’, we used locally drawn tap water by Saline Water Conversion Corporation (SWCC) who supply all of Saudi Arabia with desalinated water [36]. This will contain a ‘cocktail’ of dissolved mineral salts as background electrolytes, which is common for all environmental water samples, and generally is subject to local and seasonal variations. Table S1 (in Supplementary Section S1) illustrates the analysis of local tap water carried out by the laboratories of the ‘Water Technologies Innovation Institute & Research Advancement-Saline Water Conversion Corporation (WTIIRA-SWCC)’ [37]. However, as we will show in the context of Figure 3/Section 3.1, our sensor is robust against interference from the most common physiological ions, as they are found in potable water. To test EGFET response to H_2_S, we purchased a saturated stock solution of H_2_S in water from Ricca Chemical Company, c_stock_ = c_sat_ = 4 g/L = 117 mM. We confirmed the concentration by measuring pH, which gave the value pH = 4.0 as expected for 117 mM H_2_S_aq_ with acid dissociation constant k_a_(H_2_S) = 9.1 × 10^−8^ mole/L. For qualitative tests, we measured transfer characteristics before and after titrating (50…60) μL of a saturated H_2_S stock solution into the EDL pool, achieving a concentration of c_pool_ = c_stock_/101, which is still far greater than the potability c_pot_ = 1.5 μM. To test for interference and matrix effects from typical co-solutes in parallel to H_2_S in sewage-polluted water, we used human urine donated by one of the authors. Human urine is an aqueous ‘cocktail’ containing urea, inorganic salts, organic compounds, organic ammonium salts, and small amounts of proteins, hormones, and a wide range of metabolites [38]. However, it does not represent a similar biohazard as faeces, as urine does not carry pathogens (bacteria and viruses).

For quantitative characterisation, we first diluted stock solution down to a test solution with c_test_ = 75 μM and then titrated small aliquots of test solution into the 5 mL pool, eventually adding up to 1 mL added c_test_. This raises c_pool_ in small steps from 0 → 12 μM, overlapping with c_pot_. A transfer characteristic was recorded after every titration step.

To quantify a possible shift of transfer characteristics due to a Δ*V_Pool_*(*c*) that may develop in the pool under H_2_S at concentration c, we (somewhat arbitrarily) evaluate the CG voltage *V_CG_* at which *I_D_* reaches 0.1 mA, and evaluate the CG voltage shift Δ*V_CG_*(*c*) with Equation (2):

Δ*V_CG_*(*c*) = *V_CG_*(*I_D_* = 0.1 mA, c) − *V_CG_*(*I_D_* = 0.1 mA, c = 0). (2)

Note that Δ*V_CG_*(*c*) is named CG shift as it is evaluated from the *I_D_ vs. V_CG_* transfer characteristics. However, *V_CG_* is controlled by the SMU independent of any events in the EDL capacitor pool. Δ*V_CG_*(*c*) is in fact a shift of *V_Pool_*, Δ*V_Pool_*(*c*), *cf.* Equation (1), resulting from concentration-dependent potentials that may develop at the electrode/solution interface.

## 3. Results and Discussion

### 3.1. Qualitative Study of Gold Electrode EGFETs for H_2_S_aq_ Detection

To establish the potentiometric sensing mechanism of Au surfaces with respect to H_2_S_aq_ proposed in the introduction, we first ran a series of tests comparing LND150 EGFET characteristics at zero *vs.* very high concentration (c = 1.16 mM = 772 c_pot_) of H_2_S_aq_ in the EDL capacitor pool for different permutations of control gate (CG) and floating gate (FG) electrodes. A quantitative response characteristic (control gate voltage shift *vs.* H_2_S_aq_ concentration) with fit to a theoretical model and evaluation of limit-of-detection (LoD) was then performed on one (suitable) electrode permutation in Section 3.2.

Table 1 shows the 4 permutations of Au electrodes (believed to be sensitive to H_2_S_aq_) and Zn electrodes (believed to be insensitive to H_2_S_aq_) as CG/FG electrodes in the EDL capacitor pool:

**Table 1:** The 4 permutations of (Au/Zn) electrodes as (CG/FG) electrodes in the EGFET EDL capacitor pool. The resulting control gate shifts were evaluated from Figure 2 below, using Equation (2).

Figure 2	CG Electrode	FG Electrode	Δ*V_CG_* [mV]
**a**	Zn	Zn	0
**b**	Zn	Au	+288
**c**	Au	Zn	−283
**d**	Au	Au	0

**Figure 2:**
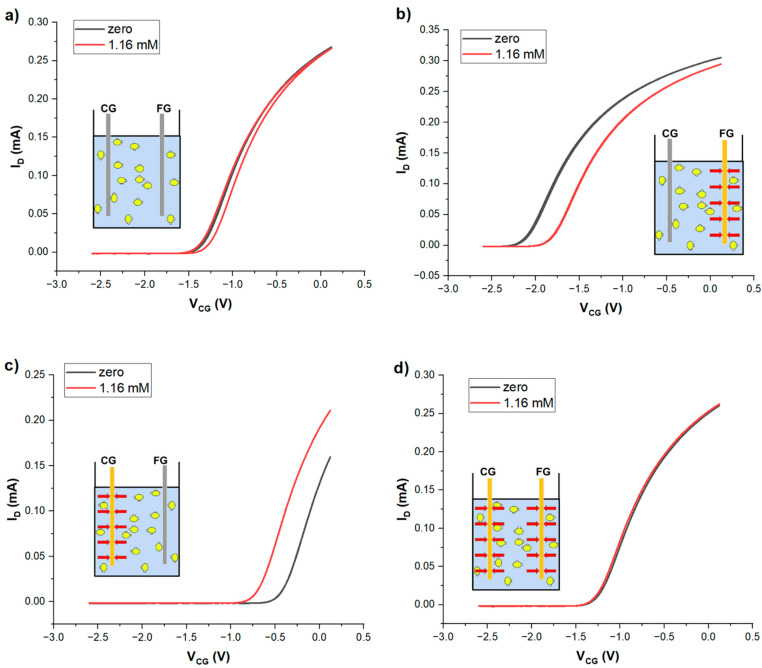
LND150 transfer characteristics (V_SD_ = +0.2 V) when gated across an EDL capacitor pool, comparing zero (black) *vs.* high (1.16 mM, red) concentration of H_2_S_aq_ in the EDL capacitor pool. (**a**) CG = FG = Zn, (**b**) CG = Zn, FG = Au, (**c**) CG = Au, FG = Zn, (**d**) CG = FG = Au. The inset to each Figure shows a schematic representation of the electrode arrangement (Zn grey, Au yellow) in the EDL capacitor pool containing water, showing dissolved H_2_S molecules (yellow/white), and the dipole moments (red arrows) developing from their adsorption on the electrode surface.

Figure 2 shows the recorded LND150 linear transfer characteristic *vs. V_CG_* under zero *vs.* 1.16 mM H_2_S_aq_.

As expected, there was no potentiometric response to H_2_S_aq_ when both CG and FG electrodes are Zn, which is not known to adsorb H_2_S_aq_. However, when either CG or FG are gold electrodes, we found a significant shift of the LND150 transfer characteristics along the *V_CG_* axis. The shift is shown in Table 1. We rationalise this shift by the selective adsorption of H_2_S onto Au, similar to the known coupling of organic thiols (R-SH) to Au surfaces, making the Au surface a sensitiser for H_2_S. The H_2_S molecule is polar (S partially negative, δ^−^, dipole moment 0.98 Debye [39]). The sorbate therefore establishes an interface potential at the (water + H_2_S_aq_) ‖ Au electrode interface that is absent at the (water + H_2_S_aq_) ‖ Zn electrode interface. However, when seen from the LND150 gate (connected to FG), the dipole direction is reversed between Zn CG/Au FG (inset Figure 2b, pointing towards FG) *vs.* Au CG/Zn FG (inset Figure 2c, pointing towards CG). Consequently, the sign of Δ*V_CG_* is also reversed, while the magnitude is very similar. The reversed dipole orientation as seen from the LND150 gate is strikingly illustrated when Au electrodes are used for both CG and FG electrodes: This leads to a cancellation of interface potentials (equal magnitudes with opposite signs), and therefore zero Δ*V_CG_*, Figure 2d. We note that the opportunity of sensing H_2_S with Au surfaces would be overlooked in an experiment where only the configuration CG = FG = Au is used.

**Figure 3:**
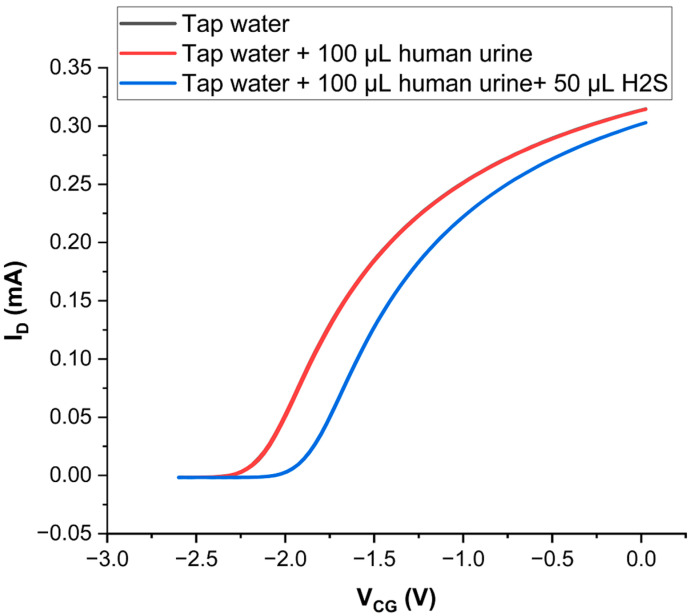
LND150 transfer characteristics (V_SD_ = +0.2 V) when gated across an EDL capacitor pool, electrodes as in Figure 2b, CG = Zn, FG = Au. Black: Tap water only in the pool. Red: After adding 100 μL of human urine. Blue: After subsequent addition of 50 μL of a saturated H_2_S_aq_ stock solution. The ‘black’ transfer is hidden under the virtually identical ‘red’ transfer.

Some watercourses may be polluted with urine but not with faecal matter, but sewage-polluted waters containing faecal matter will always contain urine in parallel. As urine does not carry the pathogens associated with faeces [38], it does not represent a similar biohazard. Urine must therefore not act as an interferent (giving a sensor response in the absence of H_2_S_aq_). On the other hand, the parallel presence of urine must not suppress the sensor’s ability to respond to H_2_S_aq_. The results of a respective test are shown in Figure 3:

Table 2 lists the typical components of urine and their concentrations:

**Table 2:** Typical composition of human urine, individual compositions may vary. The largest contribution to overall solids in urine is urea, which is not listed separately here but accounts for more than half of overall weight of dissolved solids, and most of the total nitrogen. Phosphorus (P) is largely in the form of phosphate, PO_4_^3−^, and is highly variable depending on diet. Urine contains traces of many further components, *e.g.*, hormones and metabolites. Data from [38].

Compound	c [mg/L]	Compound	c [mg/L]
N (total)	8830	Na^+^	3450
Ammonia	460	Ca^2+^	230
Nitrate/Nitrite	0.06	Mg^2+^	120
P (total)	800…2000	Cl^−^	4970
K^+^	2740	SO_4_^2−^	1500

Figure 3 shows no response of our sensor to urine pollution, the transfers shown in black and red are so similar that the ‘black’ transfer (tap water only) is hidden under the ‘red’ transfer (tap water + urine). Our frugal EGFET sensor is selective for H_2_S_aq_ over the components of urine, in particular urea, and by extension most likely over all amines. However, the presence of urine does not negate the detection of H_2_S_aq_: The addition of 50 μL of a saturated H_2_S_aq_ stock solution to a urine-polluted pool does provide a very similar response (‘blue’ transfer) as in the absence of urine, Figure 2b. Table 2 also shows that the addition of urine (even in 50-fold dilution) to our tap water adds several electrolytes in higher concentration than originally present, *cf.* Supplementary Section S1, with no impact on the recorded transfer characteristics. Our sensor is therefore robust against interference from common electrolytes, allowing application to all common water samples.

Further experiments (Figures shown as Supplementary Material) show that the addition of 50 μL more H_2_S stock solution to an EDL pool (raising c in the pool from 1.16 mM → 3.31 mM) does not lead to a further shift of *V_CG_* (Section S2, Figure S1). This suggests that the sensor response is saturated at 1.16 mM. The sensor response characteristics therefore cannot be Nernstian as Nernstian response does not saturate but shifts linearly on a log c scale with a shift of 59 mV/decade in c for monovalent ions. Nernstian characteristics are often found for potentiometric ion sensors, *e.g.*, [40,41,42], including pH-sensitive EGFETs, *e.g.*, [34]. As a small fraction of the weak acid H_2_S does dissociate in water, H_2_S + H_2_O ⇋ HS^−^ + H_3_O^+^, a Nernstian (ion-based) mechanism cannot be dismissed *a priori*. However, the saturation of the present sensor at 1.16 mM (or below) refutes a Nernstian sensing mechanism, including pH (H_3_O^+^ ion)-based response. With acid dissociation constant k_a_(H_2_S) = 9.1 × 10^−8^ mole/L, we calculate the concentration of HS^−^ as [HS^−^] = 10.3 μM at c(H_2_S) = [H_2_S] = 1.16 mM, and [HS^−^] = 16.9 μM at [H_2_S] = 3.31 mM. The same is true for the concentration of H_3_O^+^, [H_3_O^+^]. Under a Nernstian response mechanism, *V_CG_* should therefore shift by Δ*V_CG_* = 59 mV log (16.9/10.3) = 12.7 mV when raising H_2_S concentration in the EDL pool from 1.16 mM → 3.31 mM. This shift would be clearly visible on the scale of Figure S1, but it is absent. Instead, a quantitative response characteristic consistent with a non-ionic surface dipole mechanism will be established in Section 3.2. Also, when we change pH from 7 to 2 by adding a strong acid rather than H_2_S to an EDL capacitor pool, we find almost no shift in transfer characteristics, Section S3/Figure S2. A Nernstian pH sensor would respond with a *V_CG_* shift of Δ*V_CG_* = 5 pH × 59 mV/pH = 295 mV. Our EGFET is clearly sensitive to H_2_S, but not to pH, and not with a Nernstian characteristic.

When an Au contact has been used once under c = 1.16 mM, the shift in *V_CG_* is permanent. Removing a used Au electrode from 1.16 mM solution, washing in clean water, and re-immersing into an EDL pool filled with tap water free from H_2_S_aq_, leads to transfers as if the pool contained 1.16 mM H_2_S_aq_, Section S4/Figure S3. The adsorption of H_2_S onto Au leads to stable bonding of hydrogen sulphide to Au that cannot easily be washed off with water. This is not surprising since thiol-to-gold coupling leads to stable immobilisation of proteins from the aqueous phase onto Au electrodes. The ‘strength’ of the H_2_S…Au bond is quantified in Section 3.2. Used Au electrodes can therefore not be immediately reused. We attempted two recovery methods for used electrodes, ‘firing’ a used electrode in a gas flame (Section S5/Figure S4a) and washing in 1M HCl (Figure S4b). We found that firing leads to partial recovery only, apparently due to combustion of adsorbed H_2_S, but washing in 1M HCl leads to a full recovery, and even a slight improvement at positive *V_CG_*. It is known that acidification drives H_2_S out of an aqueous solution [25], apparently, it also removes it when adsorbed to an Au surface. We, therefore, recommend washing Au electrodes in 1M HCl prior to every use, including before first use, to remove any unintentionally adsorbed chemicals from its surface that possibly result from prior exposure to polluted air. The ability to fully recover a previously used Au wire for repeated use, rather than requiring a new Au electrode after every positive test, is essential to keep the promise of a ‘frugal’ sensor, as only one 2 cm long Au wire is ever required for an unlimited number of tests.

When an Au contact has been used once under c = 1.16 mM, the shift in *V_CG_* is permanent. Removing a used Au electrode from 1.16 mM solution, washing in clean water, and re-immersing into an EDL pool filled with tap water free from H_2_S_aq_, leads to transfers as if the pool contained 1.16 mM H_2_S_aq_, Section S4/Figure S3. The adsorption of H_2_S onto Au leads to stable bonding of hydrogen sulphide to Au that cannot easily be washed off with water. This is not surprising since thiol-to-gold coupling leads to stable immobilisation of proteins from the aqueous phase onto Au electrodes. The ‘strength’ of the H_2_S…Au bond is quantified in Section 3.2. Used Au electrodes can therefore not be immediately reused. We attempted two recovery methods for used electrodes, ‘firing’ a used electrode in a gas flame (Section S5/Figure S4a) and washing in 1M HCl (Figure S4b). We found that firing leads to partial recovery only, apparently due to combustion of adsorbed H_2_S, but washing in 1M HCl leads to a full recovery, and even a slight improvement at positive *V_CG_*. It is known that acidification drives H_2_S out of an aqueous solution [25], apparently, it also removes it when adsorbed to an Au surface. We, therefore, recommend washing Au electrodes in 1M HCl prior to every use, including before first use, to remove any unintentionally adsorbed chemicals from its surface that possibly result from prior exposure to polluted air. The ability to fully recover a previously used Au wire for repeated use, rather than requiring a new Au electrode after every positive test, is essential to keep the promise of a ‘frugal’ sensor, as only one 2 cm long Au wire is ever required for an unlimited number of tests.

### 3.2. Quantitative Calibration of Au Electrode EGFET for H_2_S_aq_ Sensing

**Figure 4:**
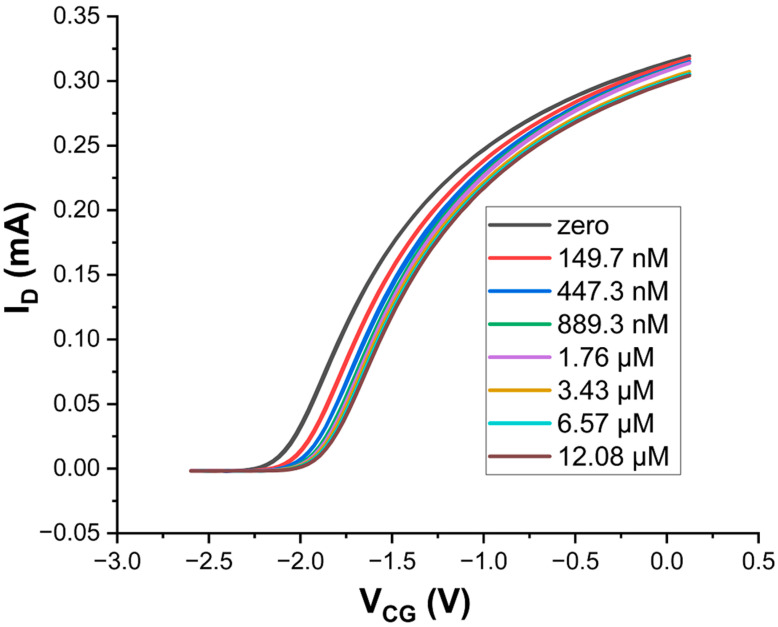
LND150 transfer characteristics (V_SD_ = +0.2 V) when gated across an EDL capacitor pool under increasing H_2_S concentration from 0 → 12 μM. CG is zinc and FG is gold.

For a quantitative calibration of our H_2_S sensors, we first diluted the stock solution down to a test solution with c_test_ = 75 μM and then titrated small aliquots of the test solution into the 5 mL pool of a Zn CG/Au FG EDL capacitor connected to LND150 gate, details in Section 2.4. We raised c_pool_ from 0 → 12 μM in small steps, overlapping with c_pot_ = 1.5 μM for H_2_S_aq_. Results are in Figure 4.

Figure 4 clearly shows a positive shift of transfers along the *V_CG_* axis with increasing concentration, c, of H_2_S_aq_. 

**Figure 5:**
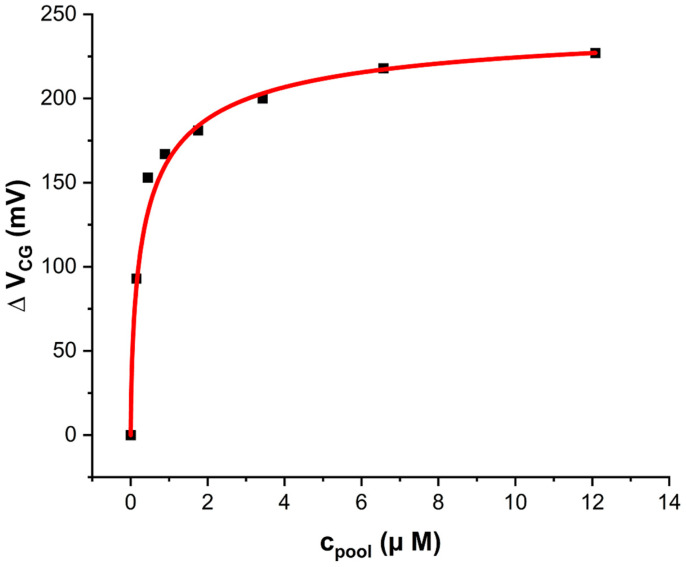
Quantitative calibration Δ*V_CG_*(*c*) of the Zn CG/Au FG EGFET sensor for H_2_S_aq_ shown in Figure 4. The red line is a fit to Equation (3).

The quantitative evaluation of Δ*V_CG_*(*c*) was as described in Section 2.4., Equation (2), and is shown in Figure 5.

Figure 5 clearly shows a sensor response to H_2_S_aq_ within the concentration test range, including a significant response at c_pot_ = 1.5 μM. Response is steep for concentrations up to ~2 μM, and approaches saturation for c > 10 μM. This type of sensor is therefore capable of detecting H_2_S_aq_ at and below potability, allowing potability decisions, see below for a simple guideline.

For fitting the observed response against a theoretical model, we have already established that the sensor response is not Nernstian, *cf.* Section 3.1 and Section S2. A common non-Nernstian response model for EGFET and WGTFT (water-gate thin film transistor) sensors is based on the Langmuir–Freundlich (LF) adsorption isotherm [33,35,43,44,45,46], Equation (3):

(3) $$∆V_{CG}\left(c\right)=∆V_{sat}\frac{{\left(kc\right)}^{\beta }}{1+{\left(kc\right)}^{\beta }}$$

The LF isotherm is a generalisation of the classic Langmuir surface adsorption isotherm, which is the special case of Equation (3) with β = 1. Δ*V_sat_* in Equation (3) is the CG shift in the limit of large analyte concentration *c*. *k* is the association constant of the analyte/sensitiser complex with units of inverse concentration, which is related to enthalpy of adsorption. Δ*V_CG_* reaches ½ Δ*V_sat_* for *c*_1/2_ = 1/*k*, regardless of β. β is a dimensionless exponent that is interpreted in terms of interactions between neighbouring adsorption sites: β = 1 (the classic Langmuir model) corresponds to the absence of such interactions, β < (>) 1 indicates the occupation of an adsorption site increases (decreases) the enthalpy of adsorption on a neighbouring site. Equation (3) is mathematically equivalent to the ‘Hill Equation’ [47] albeit the Hill equation is conventionally written in a different form. The Hill equation is routinely used to model response characteristics of biosensors, *e.g.*, [48], where β < (>) 1 is known as positively (negatively) cooperative binding. A fit to Equation (3) is shown as a solid red line in Figure 5, fit parameters are shown in Table 3:

**Table 3:** Parameters for fitting the model, Equation (3), to the EGFET response characteristics, Figure 5.

Parameter	*k* [10^6^ L/mol]	β	Δ*V_sat_* [mV]
**Value**	2.677 ± 0.54	0.66 ± 0.12	250 ± 8

Δ*V_sat_* is similar to the magnitude of Δ*V_CG_* shown in Table 1 for a far larger concentration of H_2_S_aq_, c = 1.16 mM. Association constant k leads to *c*_1/2_ = 1/*k* = 374 nM. This supports our earlier conclusion in Section 3.1 that sensor response is saturated at 1.16 mM H_2_S_aq_ = 3100 c_1/2_. The observed response characteristics allow evaluation of the limit-of-detection (LoD) of our sensor, following a procedure reported earlier (*e.g.*, [45]). This procedure is performed in Supplementary Section S6/Figure S5. We find c_LoD_ = 14.9 nM, 2 orders-of-magnitude smaller than c_pot_ for H_2_S_aq_. Our sensor is therefore capable of potability decisions even if the sample is somewhat diluted by addition to an EDL pool. Our LoD also compares favourably to some optical sensors [18,19].

To account for small differences in Δ*V_sat_* between different devices, users can always find Δ*V_sat_ a posteriori* after measuring a sample by adding an excess of H_2_S_aq_ to the EDL pool to drive the sensor into saturation. This is a variation of the ‘standard addition’ method frequently used in analytical chemistry, *e.g.*, [49]. Finding a saturated response in the order of ~250 mV or more confirms that both sensor, and operator, were capable during the prior sampling. This includes proof that previous use/cleaning in 1M HCl has not degraded Au electrodes. We then normalise Equation (3) to Δ*V_CG_*(*c*)/Δ*V_sat_* using the specific Δ*V_sat_*. Potability decisions are more robust on the normalised Δ*V_CG_*(*c*)/Δ*V_sat_* scale. With c = c_pot_ = 1.5 μM and k and **β** from Table 3, we find Δ*V_CG_*(c_pot_)/Δ*V_sat_* = 0.714, a smaller Δ*V_CG_*(c_pot_)/Δ*V_sat_* ratio indicates potability *w.r.t.* H_2_S.

## 4. Summary and Conclusions

Many communities nowadays are rightly concerned about the contamination of their water supply with faecal matter. H_2_S_aq_ is usually the result of faecal matter decomposing in water and therefore is a ‘telltale’ sign for the possible parallel presence of pathogens associated with faeces. We here present an EGFET sensor as a ‘frugal’ approach to the detection of such contamination. It is assembled from basic, cheap, and widely available components, making it adequate for use by concerned communities without reliance on government agencies, which often are underfunded, and lack expensive commercial equipment.

As an underlying sensing principle, we find that the chemical affinity of thiols to gold (Au) surfaces extends to hydrogen sulphide, H_2_S. When an Au electrode is inserted into an aqueous medium carrying dissolved H_2_S (H_2_S_aq_), H_2_S_aq_ adsorbs onto the Au surface to an extent that depends on its concentration. As the H_2_S molecule carries a dipole moment, adsorption leads to an interface potential that can be transduced into a control gate (CG) voltage shift in an extended-gate field effect transistor (EGFET) sensor setup. CG shift is absent when two Zn electrodes are used in the same EGFET setup. The sign of the observed CG shift depends on the choice of whether the Au electrode is used as the EGFET’s control gate, or its floating gate (FG). When Au is used for both CG and FG electrodes, CG voltage shift is absent again as the shifts on each electrode are equal in magnitude but opposite in sign. Human urine as an inevitable co-solute of H_2_S_aq_ from faecal pollution does not lead to false positives but does not prevent the detection of H_2_S_aq_ either. The indifference against urine also shows that our sensor will perform equally well under different ‘background’ water, as urine adds a multitude of common ions (Table 2) without detrimental effect to sensor performance.

Adsorption of H_2_S_aq_ onto Au electrodes is persistent after an electrode is removed from contaminated water, electrodes do not recover by simple washing in water. However, Au electrodes can be fully returned to their initial, sensitive state by washing in 1M HCl. Quantitatively, the sensor response saturates at a high concentration of H_2_S_aq_ with a CG voltage shift in the order (250…300) mV. Saturation contradicts an ionic (Nernstian) sensing mechanism. The quantitative sensor response CG voltage shift *vs.* H_2_S_aq_ concentration instead follows the Langmuir Freundlich (LF) adsorption isotherm that is frequently found adequate to fit the concentration dependency of dipole-based interface potentials. The limit-of-detection of the sensor described here is 2 orders of magnitude below the potability of H_2_S_aq_, making this type of sensor adequate to assess potability with respect to H_2_S_aq_.

As auxiliary equipment, practical users shall be issued with a concentrated solution of H_2_S_aq_, for *a posteriori* calibration of Δ*V_sat_*, as explained at the end of Section 3.2 and 1M HCl for cleaning and recycling Au electrodes after use. After testing a sample with the EGFET and finding a CG shift in response to its *a priori* unknown H_2_S_aq_ concentration, c_sample_, *conc*. H_2_S_aq_ shall then be added to drive the sensor into saturation. Finding Δ*V_sat_* in the expected range confirms a functioning sensor, and precise knowledge of Δ*V_sat_* for the device at hand allows potability decisions against a chart showing the normalised Δ*V_CG_*(*c*)/Δ*V_sat_* scale. A sample that shows more than the ‘critical’ response Δ*V_CG_*(*c_pot_*)/Δ*V_sat_* = 0.714 indicates that such water may carry biohazards. As an immediate measure, such water shall be boiled before consumption, followed by further analysis in a professional (chromatographic) laboratory and remediation of the origin of the H_2_S_aq_ pollution.

We have shown the selectivity of our sensor for H_2_S_aq_ over pH and urine. The urine test, Figure 3, included exposure to urea as a generic amine as well as a ‘cocktail’ of common ions. This includes sulphate, another common waterborne sulphur compound, at >300 μM (*cf.* Table 2 after 50-fold dilution). We find no ‘false positive’ from any of these potential interferants. However, testing EGFET sensors against interferants can never be fully comprehensive. An obvious interferant ‘family’ will be thiols (R-SH with R ≠ H), but these are not common water pollutants. Generally, EGFET sensors are not as robust against interference as chromatography, which is a ‘2-dimensional’ method: Solutes are first separated and identified by different elution times, then quantified by peak amplitude. In an EGFET, a single response (Δ*V_CG_*) serves to both identify, and quantify, analyte concentration. While a negative test clears a sample of H_2_S_aq_, we cannot fully exclude that a positive result may originate from an interferant. We recommend that positive samples be sent for a full test in a professional water laboratory.

While the EGFET sensor described here is itself a very cheap device, we did require a pair of source-measure units (SMUs) for the measurement of transfer characteristics and had to evaluate transfers manually to find CG voltage shifts. This procedure calls for further simplification. An operational amplifier (OpAmp) circuit for the readout of CG shift with a simple voltmeter, without the need to record full transfer characteristics, has been proposed, *e.g.*, by Bergveld [50]. An improved version of such a circuit is currently under development in our laboratory.

## Data Availability

Data are contained within the article.

## Supplementary Materials

The following supporting information can be downloaded at: https://www.mdpi.com/article/10.3390/s24020407/s1, Table S1: Local water analysis results, Figure S1: EGFET response saturation at high H_2_S concentration, Figure S2: Absence of EGFET response to pH, Figure S3: Persistence of H_2_S on Au surface, Figure S4: Re-covering used electrodes, Figure S5: Limit-of-detection (LoD).
