# Supplementary material for: A ‘Frugal’ EGFET Sensor for Waterborne H2S
Source: Sensors (Basel). 2024 Jan 9;24(2):407. doi: 10.3390/s24020407 (PMC10818413; doi:10.3390/s24020407)
Supplement: Supplementary file 1 [file sensors-24-00407-s001.zip › sensors-2790224-supplementary.pdf]

Supplementary Material

S1 Local Water Analysis Results

Table S1: Analysis of local tap water carried out by the laboratories of the 'Water Technologies Innovation Institute & Research Advancement in Saline Water Conversion Corporation (SWCC)' [37].

Parameter	Unit	Result
pH	-	8.3
Conductivity	µS/cm	235
TDS	mg/L	122
R-Cl2	mg/L	0.07
Alkalinity	mg/L	32
Hardness	mg/L	40
HCO_3^-	mg/l	39
Na^+	mg/L	16
Ca^{2+}	mg/L	15
Mg^{2+}	mg/L	2
Cl^-	mg/L	27
SO_4^-	mg/L	8

S2 EGFET response saturation at high H_2S concentration

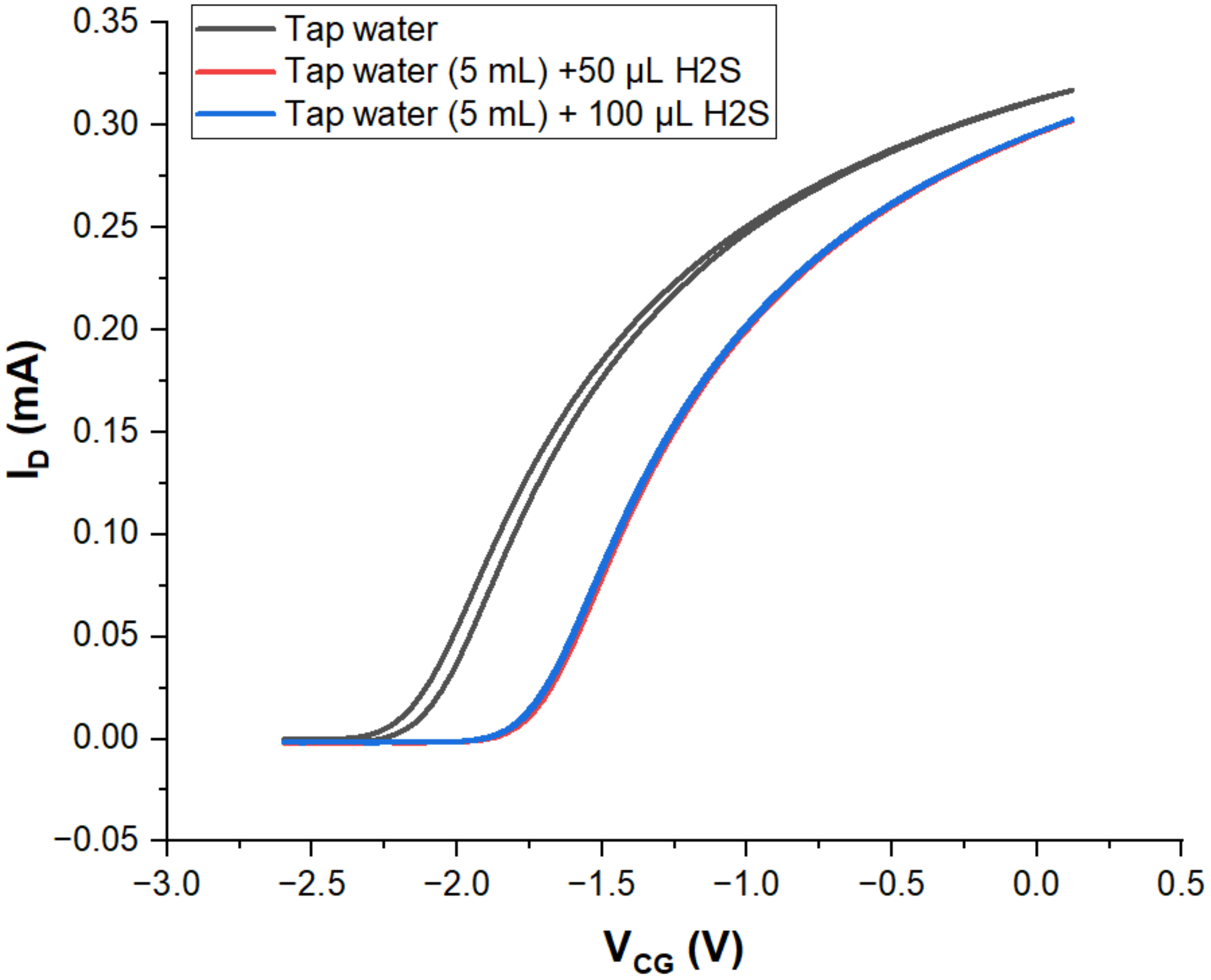

Figure S1. Comparison of transfers in an EGFET with 5 mL EDL pool filled with tap water, after adding 50 µL, and after adding 2 x 50 µL = 100 µL of H_2S stock solution at c_{stock} = 117 mM. CG was zinc, FG was gold.

Figure S1 shows that increasing H_2S concentration in the EDL pool from 0 → 1.16 mM leads to a large shift along the V_{CG} axis, but a further increase from 1.16 mM → 2.31 mM does not lead to a further shift. We conclude that the EGFET response has saturated at 1.16 mM or below.

S3 Absence of EGFET response to pH

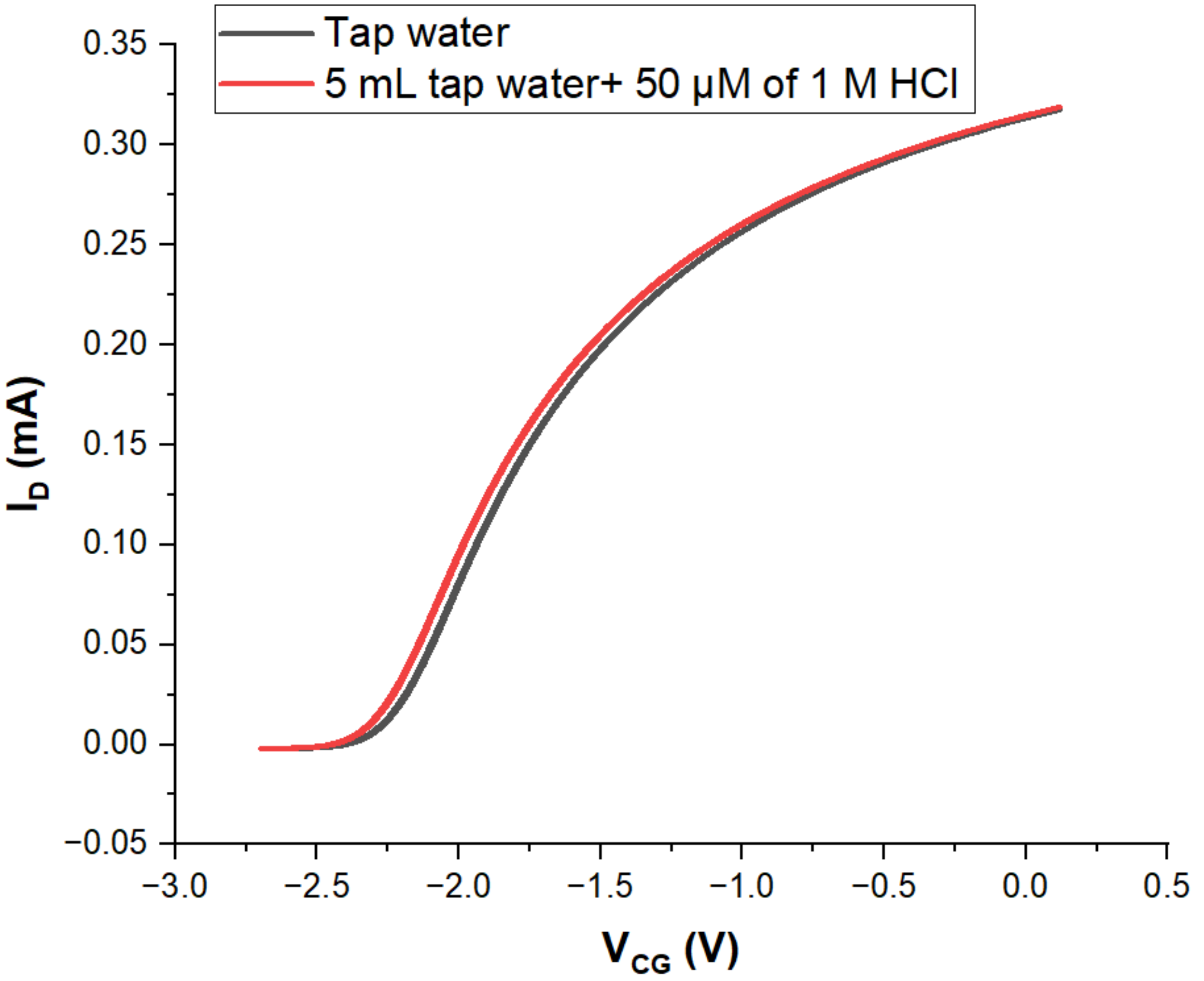

Figure S2. Comparison of transfers in an EGFET with 5 mL EDL pool filled with tap water (black), and after adding 50 µL of 1 M HCl (red). This turns the water in the pool into 10 mM HCl and as a strong acid like HCl fully dissociates, pH drops from 7 (water) to 2 (10 mM HCl) However, the transfer characteristics are very similar before and after the addition of HCl, our EGFET does not respond to pH.

S4 Persistence of H_2S on Au surface

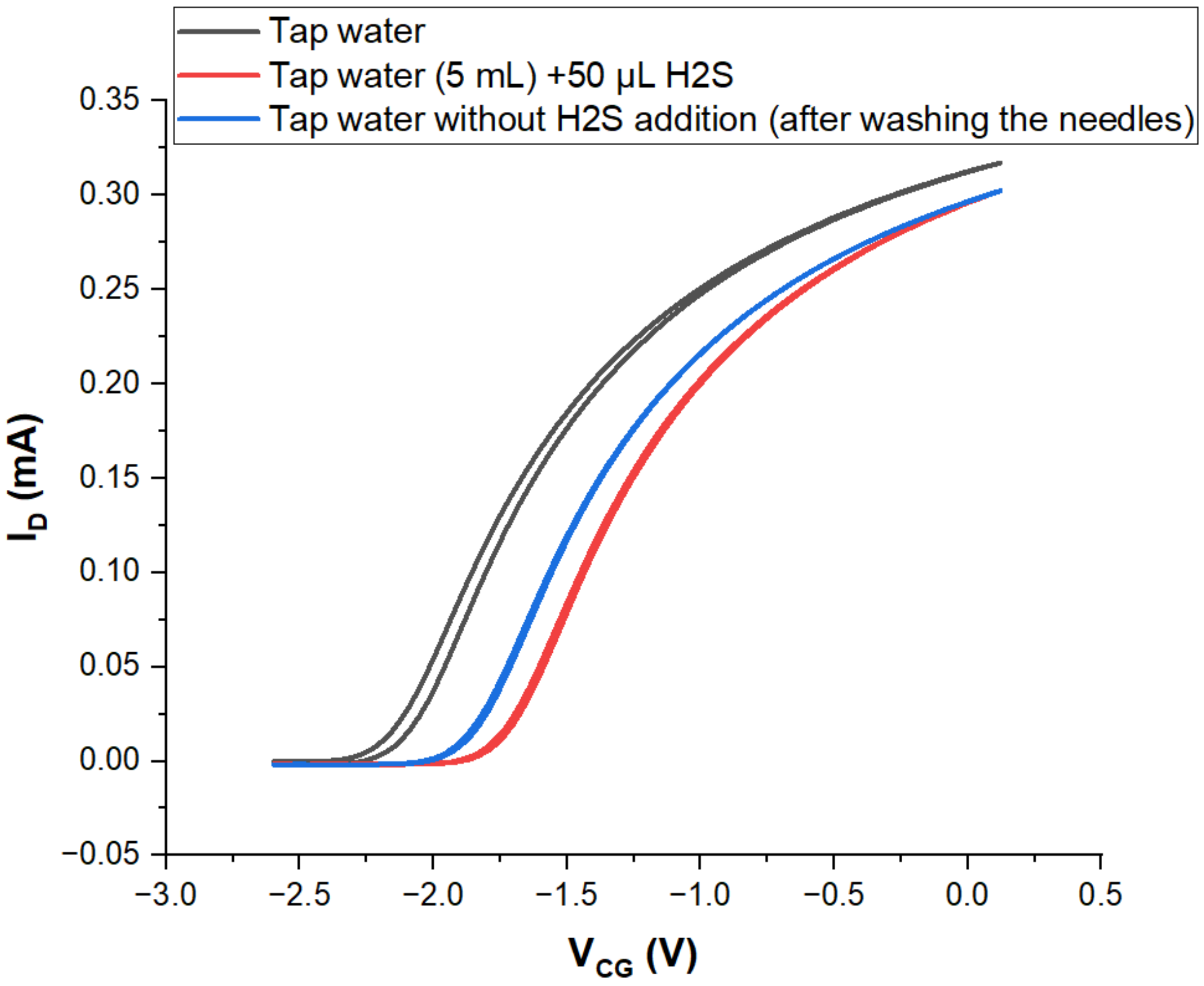

Figure S3. Comparison of transfers in an EGFET with 5 mL EDL pool filled with tap water, after the addition of 50 µL H_2S stock solution at c_{stock} = 117 mM, and after washing and re-using the same Au electrode in another EDL pool filled with tap water again, without H_2S addition. CG was zinc, FG was gold

Figure S3 shows that an Au electrode that was once exposed to H_2S retains a V_{CG} shift even after it is removed from the H_2S solution, washed in water, and then immersed into a new pool filled with fresh tap water / without H_2S. We conclude that H_2S remains adsorbed onto an Au surface even when the surface is removed from the H_2S solution and washed. Electrodes cannot be re-used without further measures.

S5 Recovering used electrodes

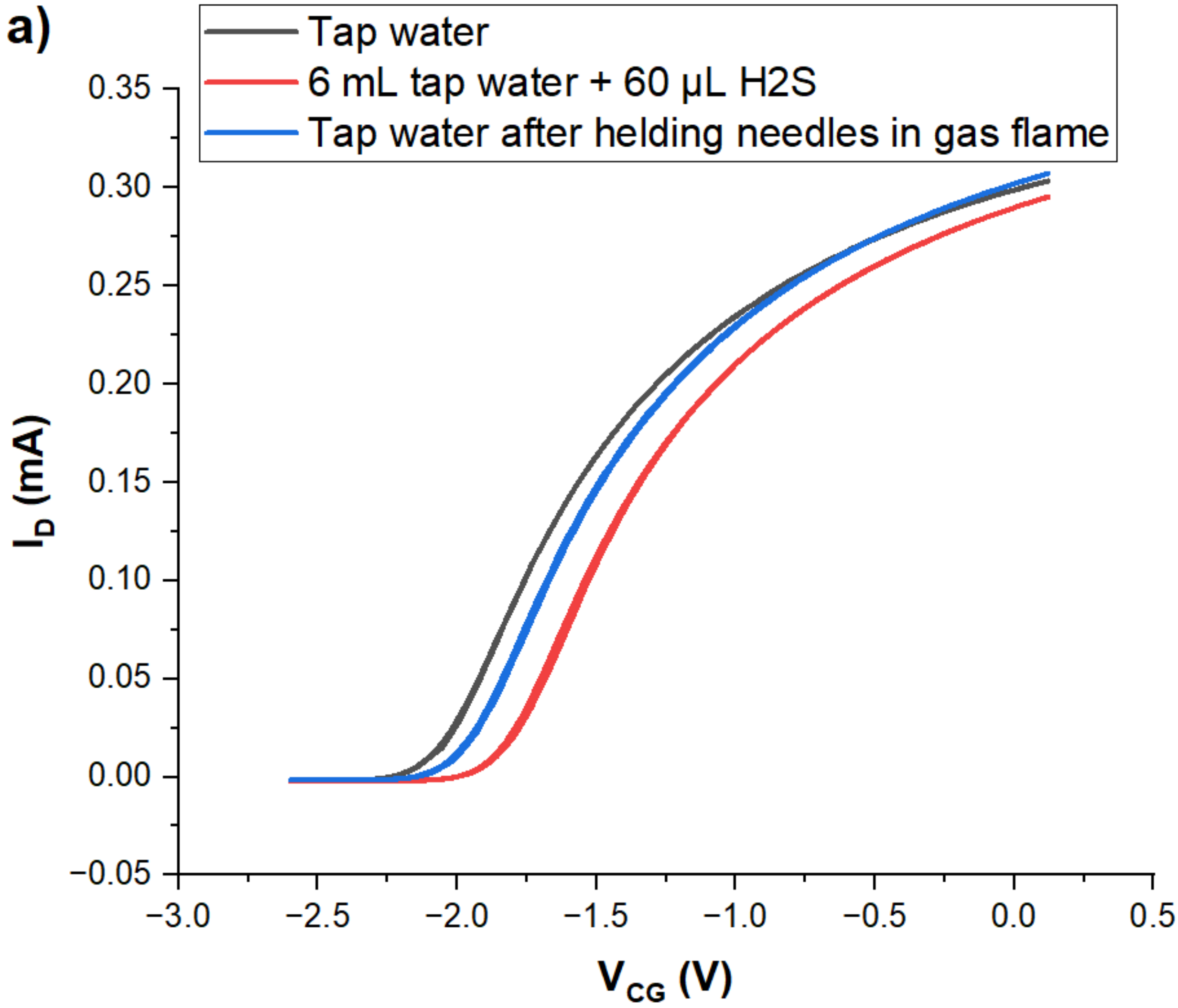

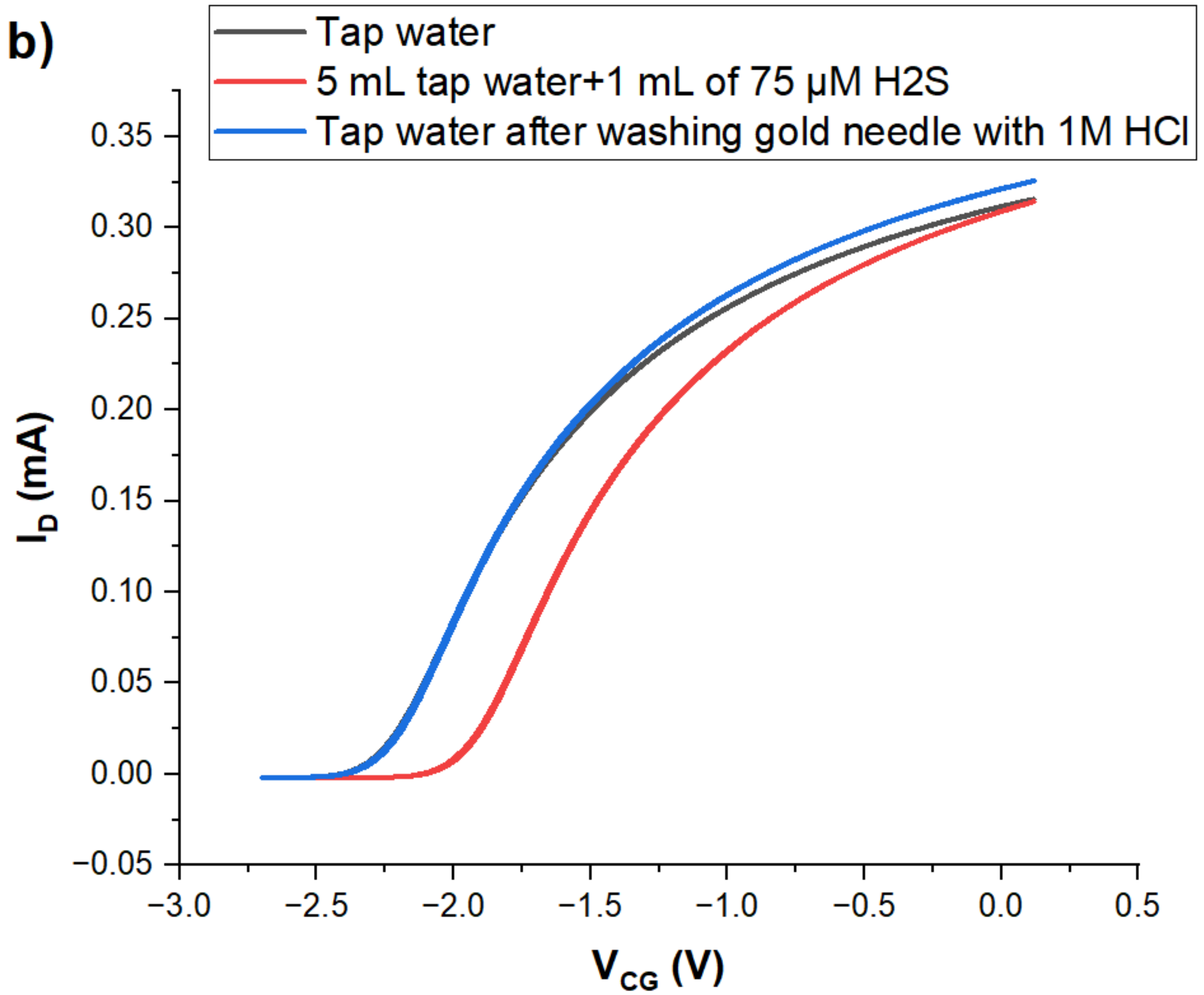

Figure S4. Comparison of transfers in an EGFET with 6 mL EDL pool filled with tap water (black), after the addition of 60 µL H_2S stock solution at c_{stock} = 117 mM (red), and after different attempts at recovering the initial performance in tap water (without H_2S) again (blue).CG was zinc, and FG was gold. **a)** 'firing' an exposed needle in a gas flame, and re-using the same Au electrode in another EDL pool filled with tap water again, without H_2S addition. **b)** washing an exposed needle in 1M HCl.

Figure S4a shows that an Au electrode that was once exposed to H_2S but then removed from the solution, dried, and held into a gas flame does partly recover when re-used in a new pool that contains only 'clean' tap water without added H_2S. However, the electrode retains a small V_{CG} shift compared to its pristine state. However, washing a used needle in 1M HCl does completely recover the original transfer.

S6 Limit-of-detection (LoD)

The limit-of-detection (LoD) of our EGFET sensor can be evaluated from the response characteristic shown in Figure 5, with the help of the fit parameters in Table 3 for the fit of Figure 5 to Equation (3). The data in Figure 5 are first re-plotted in linearised form, $((k\ c_{pool})^{\beta} + 1)\ \Delta V_{CG}$ *vs.* $(k\ c_{pool})^{\beta}$, Figure S5.

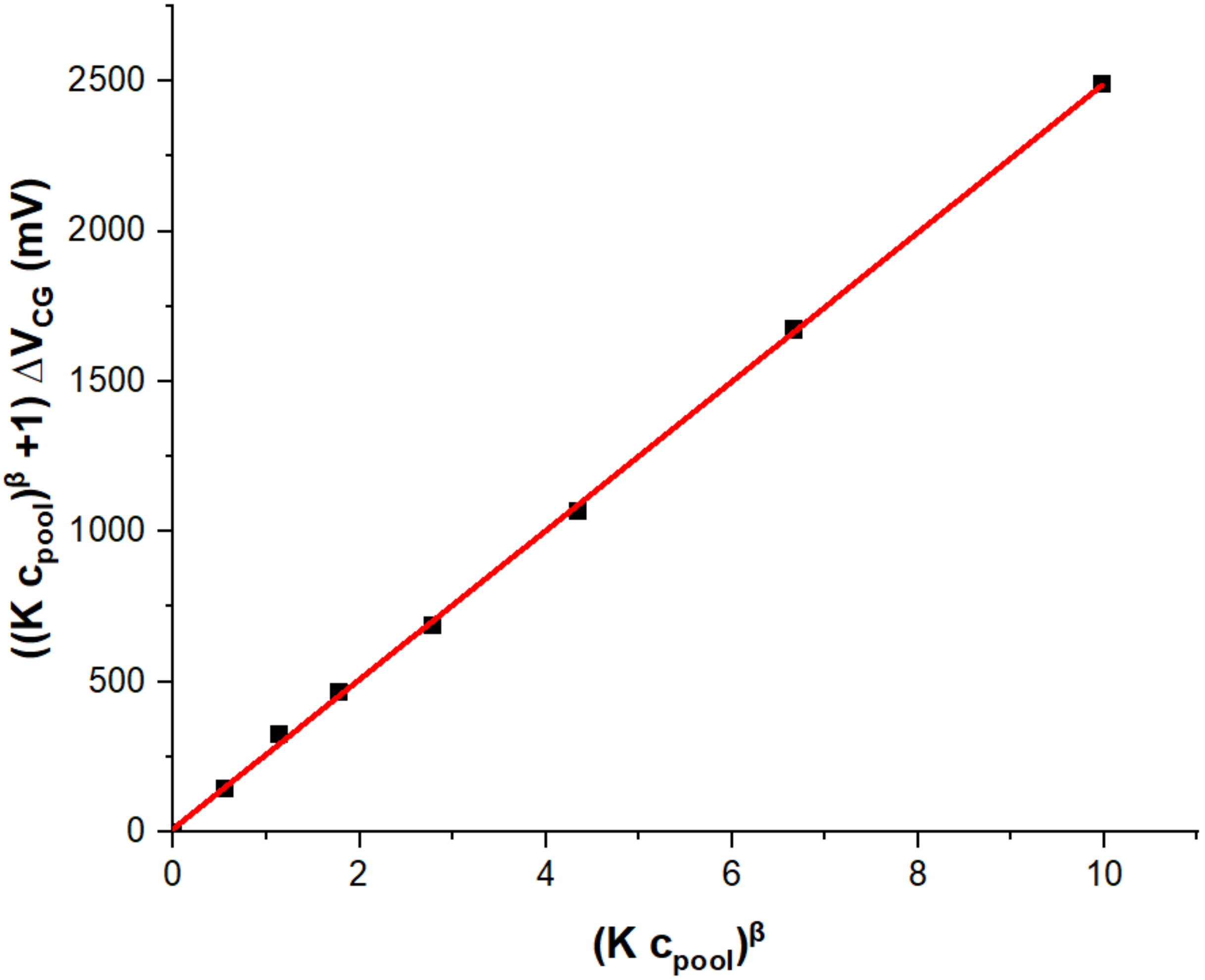

Figure S5. Linearised plot of the data in Figure 5, $((k\ c_{pool})^{\beta} + 1)\ \Delta V_{CG}$ *vs.* $(k\ c_{pool})^{\beta}$ using the parameters k and **β** listed in Table 3. Also shown in red is a fitted straight line.

A straight line of the form Equation (S1) is fitted to the linearised plot:

$$((k\ c_{pool})^{\beta} + 1)\ \Delta V_{CG} = m(k\ c_{pool})^{\beta} + b \qquad \text{(S1)}$$

With slope m and intercept b. We find fit parameters m +/- Δm= (248.1 +/- 2.1) mV and b +/- Δb = (11.9 +/- 9.8) mV with Δm and Δb the 'estimated standard errors' (e.s.e's) of m and b. Δb is similar in magnitude to b itself, which is consistent with the expectation b = 0. LoD is calculated with the commonly used '3 errors' criterion,

$$(k\ c_{LoD})^{\beta} = 3\Delta b/m \quad \text{(S2)}$$

We find c_{LoD} = 14.9 nM
